# The osteology of the wrist of* Heyuannia huangi* (Oviraptorosauria) and its implications for the wrist folding mechanism

**DOI:** 10.7717/peerj.17669

**Published:** 2024-07-10

**Authors:** Rui Qiu, Yanli Du, Zhiqing Huang, Xufeng Zhu, Xiaoli Yang, Qiang Wang, Xiaolin Wang

**Affiliations:** 1 National Natural History Museum of China, Beijing, China; 2 Heyuan Museum (Heyuan Key Laboratory of Paleontological Research and Conservation), Heyuan, Guangdong, China; 3 Key Laboratory of Vertebrate Evolution and Human Origins of Chinese Academy of Sciences, Institute of Vertebrate Paleontology and Paleoanthropology, Chinese Academy of Sciences, Beijing, China; 4 University of Chinese Academy of Sciences, Beijing, China; 5 Centre for Research and Education on Biological Evolution and Environment, Nanjing University, Nanjing, Jiangsu, China

**Keywords:** *Heyuannia huangi*, Oviraptorosauria, Wrist, Ulna, Semilunate carpal

## Abstract

The wrist of extant birds is highly specialized which permits folding of the forelimb in order to protect the pennaceous feathers when they are relaxed. This mechanism is absent in most non-avian theropods and is unknown in oviraptorosaurs because of the rarity of the specimens with well-preserved wrist. Here we give a detailed description of the wrist of two three-dimensionally preserved oviraptorosaurian *Heyuannia huangi* specimens from the Upper Cretaceous in Southern China. *Heyuannia huangi* possesses a highly specialized wrist with a strongly dorsoventrally compressed distal ulna, a larger radiale angle and a strongly convex semilunate carpal. The morphology of its wrist suggests that the distal ulna would not hinder the rotation of the manus, resulting in the smallest angle between the manus and the ulna being less than 90 degrees. The combination of the morphology of the wrist of oviraptorosaurs and the phylogenetic result indicates functional convergence in the wrist of oviraptorids and extant birds.

## Introduction

**Figure 1:**
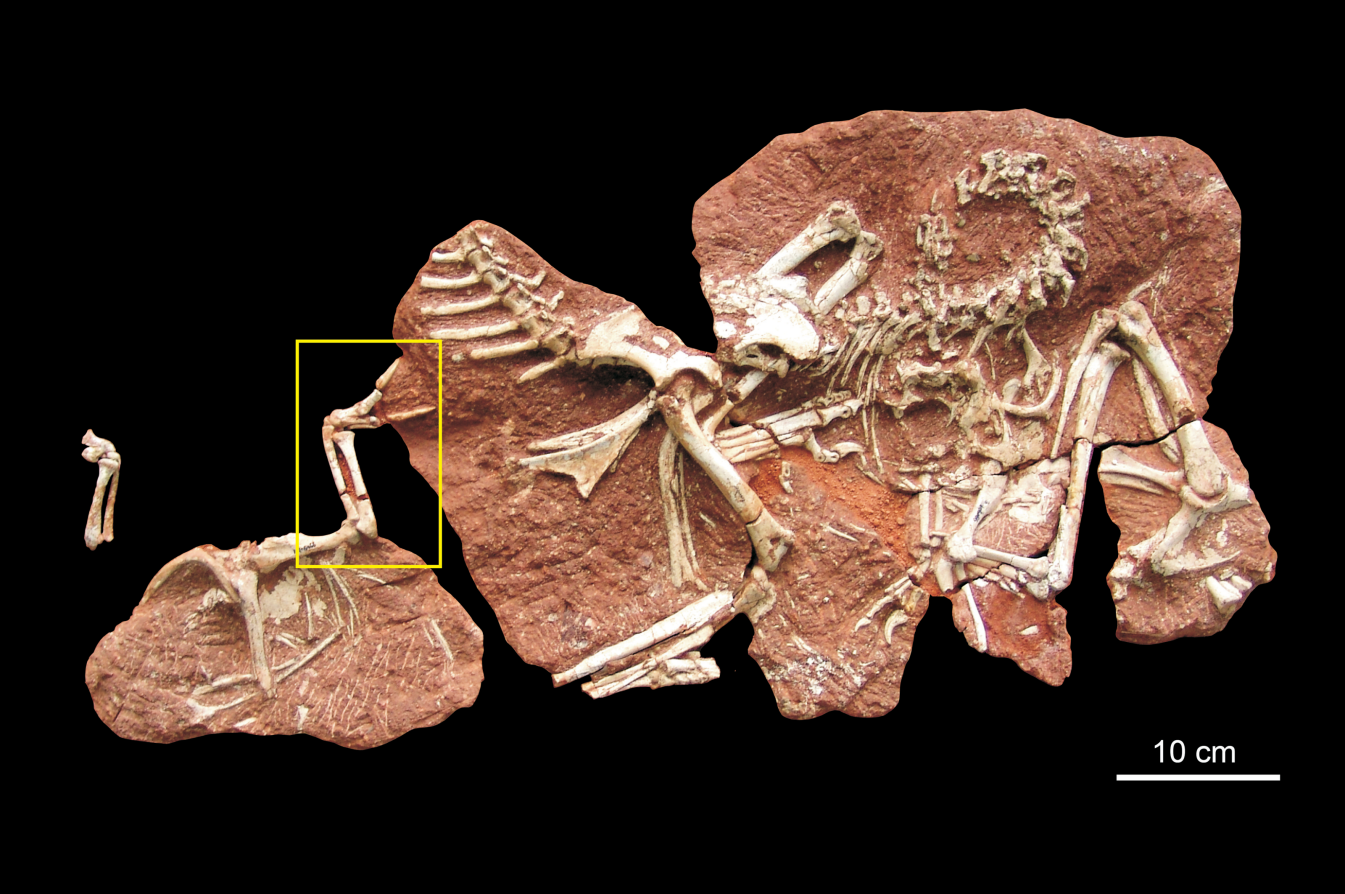
Holotype block of *Heyuannia huangi*. The yellow box indicates the forelimb in Fig. 2.

*Heyuannia huangi* (Fig. 1) is an oviraptorosaurian theropod found from the Upper Cretaceous Zhutian Formation in the Heyuan Basin of Guangdong Province, China (Lü, 2005). Zhutian Formation was initially named the Dalangshan Formation and was regarded as the Maastrichtian (Lü, 2002). The subsequent research revised these deposits to Zhutian Formation, and its age was changed to Upper Campanian (Lü, 2005).

Oviraptorosauria is a clade among non-paravian theropods that shares several morphological characters with primitive birds, including the fused dentary and premaxilla, narrow and rod-shaped jugal, loss of the maxillary and dentary teeth, ectopterygoid connecting lacrimal to the palatine, and reduction in the number of caudal vertebrae (Elzanowski, 1999; Maryańska, Osmólska & Wolsan, 2002; Lü et al., 2002). The early phylogenetic analyses performed on theropods even recovered oviraptorosaurs as flightless birds rather than non-avian theropods (Maryańska, Osmólska & Wolsan, 2002; Lü et al., 2002). Recent studies on coelurosaurian phylogeny have identified oviraptorosaurs as the basal pennaraptorans (Brusatte et al., 2014; Lee et al., 2014). Oviraptorosaurs are found to be more closely related to birds than most theropods, with the exception of deinonychosaurs. Oviraptorosaurs are regarded as feathered dinosaurs because of the presence of elongated pennaceous feathers covering on the forelimb and tail in the basal species (Ji & Ji, 1997; Ji et al., 1998; Zhou & Wang, 2000; Zhou & Wang, 2000; Qiu et al., 2019) and the presence of feather quill knobs on the ulna of the derived species (Funston & Currie, 2016). In order to protect the pennaceous feathers on the forelimb when on the ground, extant volant birds have developed a highly specialized wrist with an increased range of abduction of the manus (Sullivan et al., 2010; Hutson & Hutson, 2014). While the flexibility of the wrist compared to the extant birds has been discussed in some theropods (Gishlick, 2001; Carpenter, 2002; Senter & Robins, 2005; Senter, 2006), there are no studies on the shape and function of the wrist of oviraptorosaurs.

The well-preserved wrist in *Heyuannia huangi* provides a valuable example for studying the morphology and function of the wrist in the heyuannine oviraptorids. Although the wrist of *Heyuannia huangi* was briefly described by Lü (2002) and a relatively detailed description was given by Lü (2005), the function related to the morphology of the wrist has not been thoroughly studied. In this study, we provide a detailed description of the osteology of the wrist of *Heyuannia huangi* and examine the function of the wrist by carefully comparing its morphology with that of other pennaraptorans with the completed wrist preserved.

## Materials & Methods

All the specimens studied here were found from the Upper Cretaceous of Heyuan Basin in Heyuan, Guangdong Province, China. These specimens are housed in Heyuan Museum, a non-profit museum dedicated to research and education, established in 1982. *Heyuannia huangi* was discovered in 1999 and was the first dinosaur found in Heyuan, with its specimens becoming the initial dinosaur collections of Heyuan Museum. Since then, Heyuan Museum has become a center for the study of the dinosaurs from Southern China. A key laboratory of paleontological research and conservation is affiliated with Heyuan Museum, and all its collections are always available for research.

**Figure 2:**
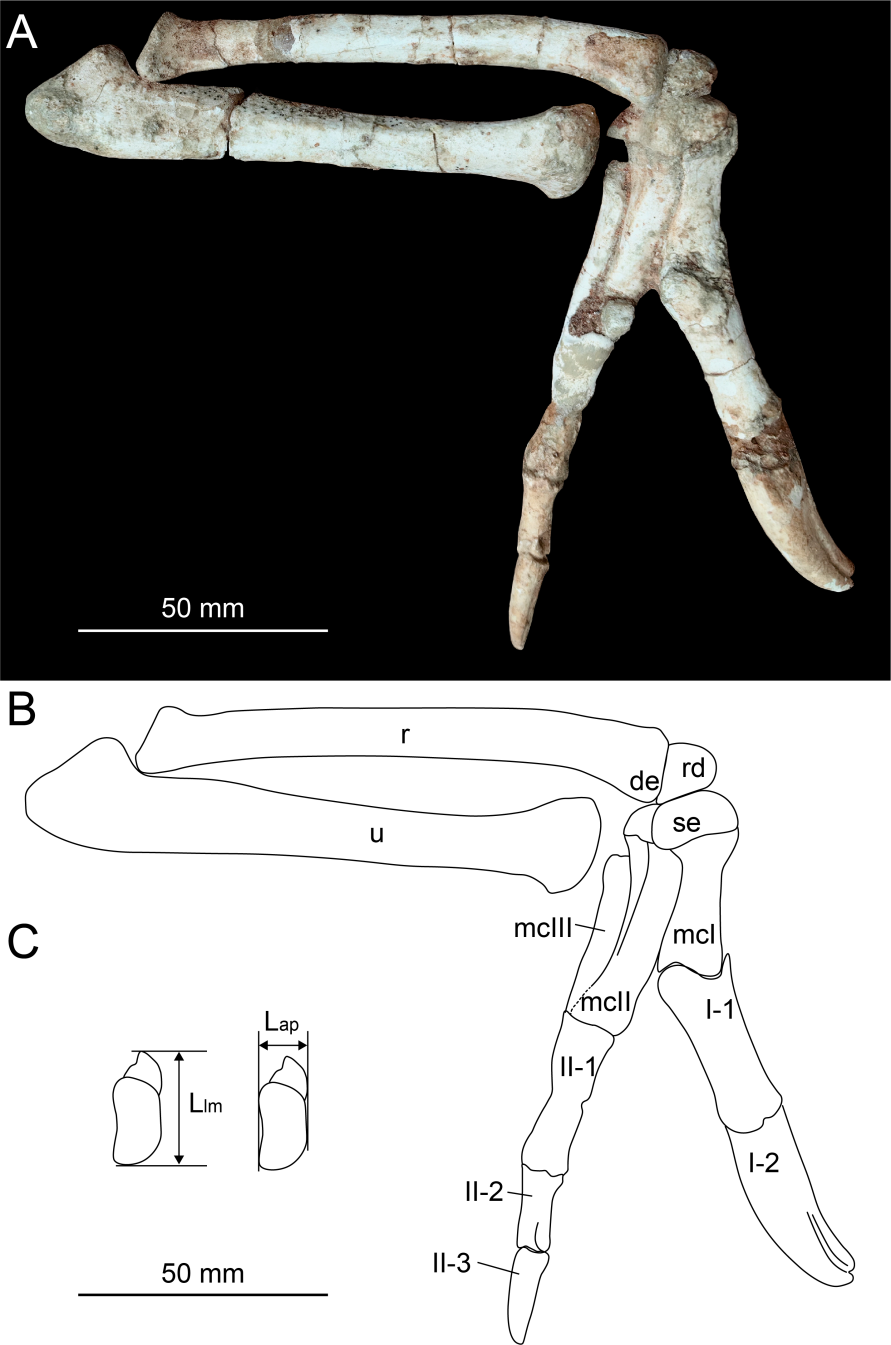
Photograph (A) and line drawing (B) of the right forelimb of *Heyuannia huangi* (HYMV 1–2) in the lateral view, and the measurements of the semilunate carpal (C). Abbreviations: de, distal expansion of radius; Lap, anteroposterior length of the semilunate carpal; Llm, lateromedial length of the semilunate carpal; mcI, metacarpal I; mcII, metacarpal II; mcIII, metacarpal III; r, radius; rd, radiale; se, semilunate carpal; u, ulna.

**Figure 3:**
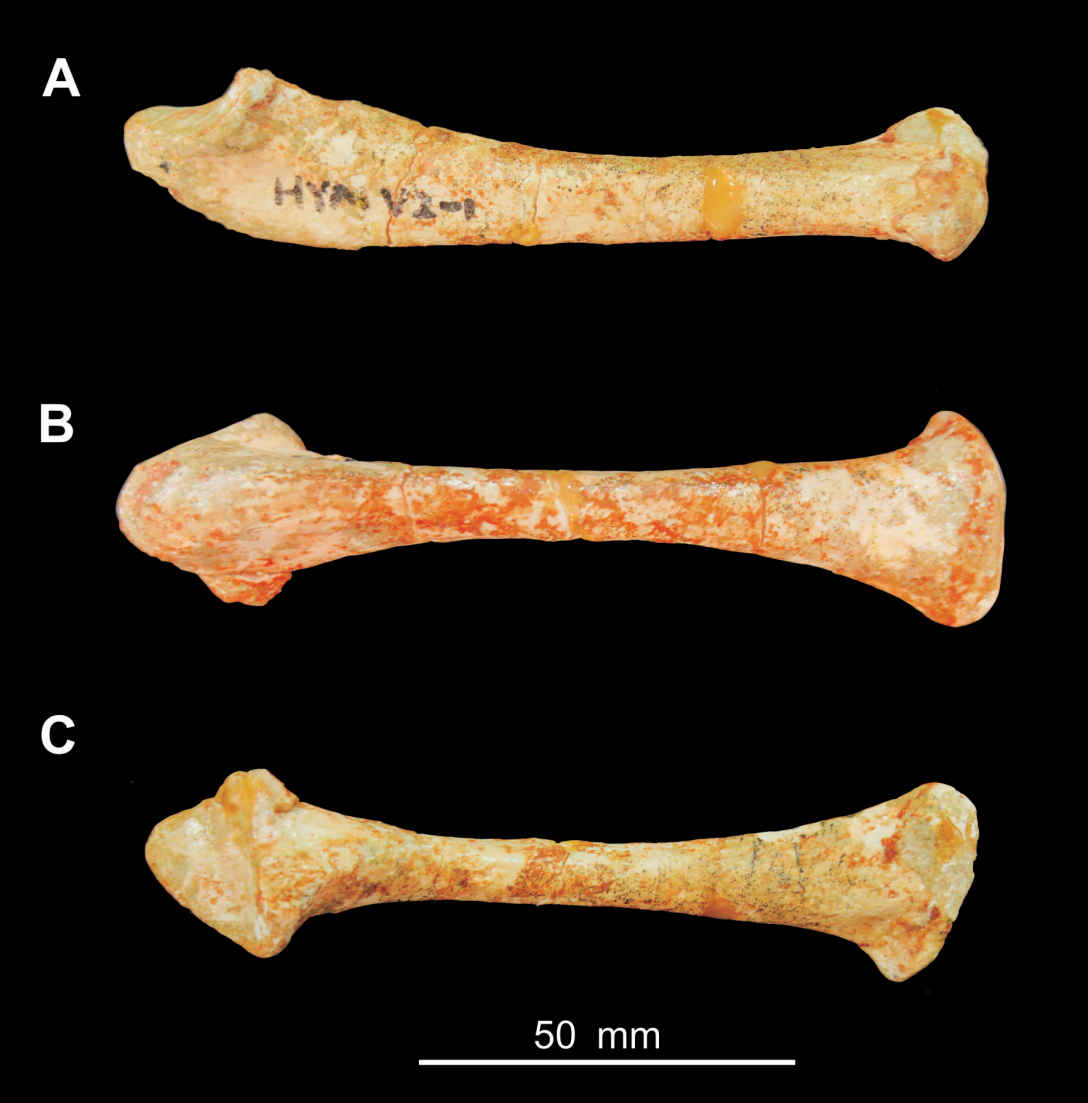
Photograph of the ulna of *Heyuannia huangi* (HYMV 2–8) in lateral view (A), ventral view (B) and dorsal view (C).

The description provided in this study is based on the nearly completed preserved right forelimb in HYMV (Heyuan Museum) 1–2 (Fig. 2) of *Heyuannia huangi*. The preserved elements include humerus, ulna, radius, radiale, semilunate carpal, and metacarpal I–III. This specimen has been fixed on the showcase of Heyuan Museum and only the lateral view of the right forelimb could be observed. Additionally, an isolated ulna HYMV 2–8 (Fig. 3) is also included in the analysis. It could be assigned to *Heyuannia huangi* because it was found from the same quarry as HYMV 1–2 in Heyuan basin and it shares the similar shape of the ulna with HYMV 1–2, especially the dorsal and ventral margin of olecranon process forming a sharp angle. Although Botelho et al. (2014) proposed the term “scapholunare” to describe the composite bone formed from the fusion of the radiale and intermedium in birds and non-avian coelurosaurian theropods, this article continues to use radiale rather than scapholunare for simplicity and consistency of comparison with other coelurosaurian research.

In order to discuss the change of the wrist during the evolution of oviraptorosaurs, a phylogenetic analysis was performed using the software package TNT 1.5 (Goloboff & Catalano, 2016) on a recently published matrix of oviraptorosaurs (Wei et al., 2022) with three new characters related to the morphology of the wrist and two additional characters related to the morphology of the manus based on Qiu et al. (2019). We used the “New Technology” search options, with sectorial search, ratchet, tree drift and tree fusion, recovering a minimum tree length in ten replicates. The rogue taxa were identified automatically with prunnelsen in TNT (Goloboff, Farris & Nixon, 2008). The data matrix is available in the Supplemental Information.

The potential range of motion of the manus was evaluated by comparing the shape of the articular faces of the carpals and distal forearm of *Heyuannia huangi* with those of whose movement of the manus has been studied in detail using bone-on-bone approach (Gishlick, 2001; Carpenter, 2002; Senter & Robins, 2005; Senter, 2006; Hutson & Hutson, 2014). This analysis method is adopted because the articular faces on the carpals of *Heyuannia huangi* show no significant difference in shape compared to those of other non-avian pennaraptorans. All pennaraptorans possess a trochlear groove along the proximal side of the proximal semilunate carpal (Ostrom, 1969; Lü, 2002; Burnham, 2004; Xu, Han & Zhao, 2014) and a concave proximal surface on the radiale (Ostrom, 1969; Burnham, 2004; Longrich, Currie & Dong, 2010; Balanoff & Norell, 2012; Tsuihiji et al., 2016). So, the discussion of the factors influencing the range of motion in the radial abduction-ulnar adduction of the manus in different non-avian pennaraptorans mainly focuses on the shape and relative size of the carpal bones and distal forearms. The maximum ulnar adduction of the hand is determined by two factors: the semilunate carpal not being dislocated from the radiale and the metacarpals not making contact with the forearm during the movement. The estimation of the angle of hand folding is made without considering the movement of the radius in all species discussed here. Since the soft tissue is not preserved in all known *Heyuannia huangi* specimens and in order to facilitate comparison with previous studies about the range of motion of the forelimb (Gishlick, 2001; Carpenter, 2002; Senter & Robins, 2005; Senter, 2006; Hutson & Hutson, 2014), the influence of soft tissue was not taken in this study.

In order to evaluate potential range of motion of the manus, the radiale angle is measured. The radiale angle is defined as the angle between the articular surface for the radius and semilunate carpal, as described by Sullivan et al. (2010). The assessment of whether the semilunate carpal is strongly convex involves multiplying the ratio of the anteroposterior length to the lateromedial length of the semilunate carpal (Fig. 2C) by the ratio of the combined widths of the proximal articular surfaces of the first and second metacarpals to the lateromedial length of the semilunate carpal (Table 1).

## Results

### Systematic paleontology

**Table:** 

Dinosauria, Owen, 1842
Theropoda, Marsh, 1881
Maniraptora, Gauthier, 1986
Oviraptorosauria, Barsbold, 1976
Oviraptoridae, Barsbold, 1976
Heyuanninae, Yun, 2019
*Heyuannia huangi* Lü, 2002

**Locality and Horizon:** Zhutian Formation, Late Cretaceous, Campanian; Huangsha village, Heyuan City, Guangdong Province, China.

**Revised** **Diagnosis:** A oviraptorid dinosaur that can be distinguished from other oviraptorids by a unique combination of characters: quadratojugal articular surface of the quadrate more groove-like; the length of dentary subequal to the length of surangular, the external mandibular fenestra locating at the middle of mandible (sharing with *Yulong*); pneumatic foramina present on the cervical ribs; olecranon process development, the dorsal and ventral margin of olecranon process forming a sharp angle (which is right angle in other caenagnathoids); metacarpal I longer than half the length of metacarpal II; manual phalanx II-1 longer than II-2. It can be distinguished from *Heyuannia yanshini* by dorsal margin of ilium arched; pubis subequal to the length of ischium.

**Table 1:** The parameters of the semilunate carpal. The result multiplying the ratio of the anteroposterior length to the lateromedial length of the semilunate carpal (Rll) by the ratio of the combined width of the proximal articular surfaces of the first and second metacarpals to the lateromedial length of the semilunate carpal (Rwl) indicates the convexity and relative size of the semilunate carpal. The taxa with result less than 0.4 are scored as 0 in Character 251. The taxa with result more than 0.4 are scored as 1 in Character 251. The Rll and Rwl are from measuring the high-resolution images and detailed description except *Heyuannia huangi*.

	R_ll_	R_wl_	R_ll∗_ R_wl_	Image or description reference
Taxa included in the phylogenetic analysis in this article				
*Herrerasaurus*	0.330239	0.842458	0.278212	Xu, Han & Zhao (2014)
*Archaeopteryx*	0.648590	1.000000	0.648590	Wellnhofer (2009)
*Xingtianosaurus*	0.440284	0.768116	0.338189	Qiu et al. (2019)
*Caudipteryx*	0.422515	0.517697	0.218735	Zhou et al. (2000)
*Hagryphus*	0.435163	1.000000	0.435163	Zanno & Sampson (2005)
*Khaan*	0.425841	1.000000	0.425841	Balanoff & Norell (2012)
*Heyuannia huangi*	0.512051	1.000000	0.512051	This article
*Oksoko*	0.816154	0.783495	0.639453	Funston et al. (2020)
*Heyuannia yanshini*	0.484362	1.000000	0.484362	Easter (2013)
Taxa from other coelurosaurian lineages				
*Guanlong*	0.621703	0.466353	0.289933	Sullivan et al. (2010); Xu, Han & Zhao (2014)
*Deinonychus*	0.441348	1.000000	0.441348	Ostrom (1969)
*Alxasaurus*	0.390381	0.914634	0.357056	Sullivan et al. (2010); Xu, Han & Zhao (2014)
*Huaxiagnathus*	0.378141	0.919268	0.347613	Botelho et al. (2014)

### Description of the wrist

The distal end of radius is ventrally expanded (Fig. 2) as in *Machairasaurus* (Longrich, Currie & Dong, 2010), different from a dorsoventral expansion in *Anzu* (Lamanna, Sues & Schachner, 2014), *Nemegtomaia* (Fanti, Currie & Badamgarav, 2012), *Khaan* (Balanoff & Norell, 2012) and *Citipati* (Norell et al., 2018), or the absence of obvious expansion in caudipterids (Zhou & Wang, 2000; Zhou & Wang, 2000; Qiu et al., 2019). The distal end of radius is triangular in the distal view. The distal end of ulna is strongly dorsoventrally compressed and has a plate-like shape (Fig. 3B), similar to other caenagnathoids with well-preserved distal ulna, such as *Khaan* (Balanoff & Norell, 2012), Nemegtomaia (Fanti, Currie & Badamgarav, 2012), *Oksoko* (Funston et al., 2020), *Citipati* (Norell et al., 2018), *Anzu* (Lamanna, Sues & Schachner, 2014) and *Yulong* (Wei et al., 2022). The distal ulna of caudipterids possesses no compression (Zhou & Wang, 2000; Zhou & Wang, 2000; Qiu et al., 2019). There are two carpals preserved. The radiale is trapezoid (Fig. 2), and its proximal surface is generally concave for the contact with the radius. The radiale angle is approximately 58 degrees, similar to most oviraptorosaurs but larger than other non-avian theropods (Sullivan et al., 2010; Qiu et al., 2019). The semilunate carpal is nearly twice the size of radiale as in *Caudipteryx* and *Oksoko* (Zhou & Wang, 2000; Funston et al., 2020). The semilunate carpal is nearly triple the size of radiale in *Hagryphus* (Zanno & Sampson, 2005). The radiale and semilunate carpal share a similar size in most oviraptorosaurs with known radiale and semilunate carpal, (Longrich, Currie & Dong, 2010; Balanoff & Norell, 2012; Qiu et al., 2019). In the lateral view, the length of the proximal margin of the radiale in *Heyuannia huangi* is subequal to the height of the distal end of the radius. However, the proximal margin of the radiale is smaller than half the height of the distal end of the radius in the lateral view in *Caudipteryx* and *Oksoko*. These conditions suggest that the semilunate carpal is larger than radiale in *Caudipteryx* and *Oksoko* due to the strong reduction of the radiale, rather than the enlargement of the semilunate carpal as in *Heyuannia huangi*. The semilunate carpal is strongly convex. The ratio between the anteroposterior length and the lateromedial length is approximately 0.51, subequal to that of *Heyuannia yanshini* (0.49). This ratio is larger than those of most oviraptorosaurs (0.44 in *Xingtianosaurus*, 0.42 in *Caudipteryx*, 0.44 in *Hagryphus*, 0.43 in *Khaan*), and smaller than that of *Oksoko* (0.82). As in other pennaraptorans, a deep transverse groove runs along the entire arc of the proximal semilunate carpal (Xu, Han & Zhao, 2014), offering a gliding articular surface for the radiale. The gliding articular surface for the radiale is symmetrical, differs from an asymmetrical gliding arc for the radiale in *Hagryphus* (Zanno & Sampson, 2005). The distal surface of the semilunate carpal covers the proximal end of metacarpal I and metacarpal II, as in other caenagnathoids except *Oksoko* (Zanno & Sampson, 2005; Longrich, Currie & Dong, 2010; Balanoff & Norell, 2012; Fanti, Currie & Badamgarav, 2012; Funston et al., 2020), which means the ratio of the combined widths of the proximal articular surfaces of the first and second metacarpals to the lateromedial length of the semilunate carpal is 1. In *Oksoko*, the semilunate carpal covers only half of the proximal end of metacarpal I and metacarpal II (Funston et al., 2020), and the ratio of the combined widths of the proximal articular surfaces of the first and second metacarpals to the lateromedial length of the semilunate carpal is 0.78. In contrast to the description from Lü (2005), the semilunate carpal is not fused with metacarpal I and metacarpal II. An obvious suture between the semilunate carpal and the first two metacarpals is present.

### Phylogenetic analysis

The phylogenetic position of *Heyuannia huangi* has been analyzed in several articles and all of which recovered it as a member of the Heyuanninae (Funston & Currie, 2016; Lü et al., 2016; Lü et al., 2017; Qiu et al., 2019; Funston et al., 2020). However, the data matrices of previous studies lack the morphological characters related to the change of the wrist. In order to analyze the evolution of the wrist in this clade, three additional characters related to the change of the wrist are added in the new data matrix:

Character 249. Distal end of ulna: (0) not strongly dorsoventrally compressed; (1) strongly dorsoventrally compressed.

Character 250. Radiale angle: (0) less than 40 degrees; (1) 40 degrees or greater.

Character 251. Semilunate carpal, the product of multiplying the ratio of the anteroposterior length to the lateromedial length of the semilunate carpal by the ratio of the combined widths of the proximal articular surfaces of the first and second metacarpals to the lateromedial length of the semilunate carpal: (0) <0.4, semilunate carpal not strongly convex and relatively small; (1) >0.4, semilunate carpal strongly convex and relatively enlarged.

**Figure 4:**
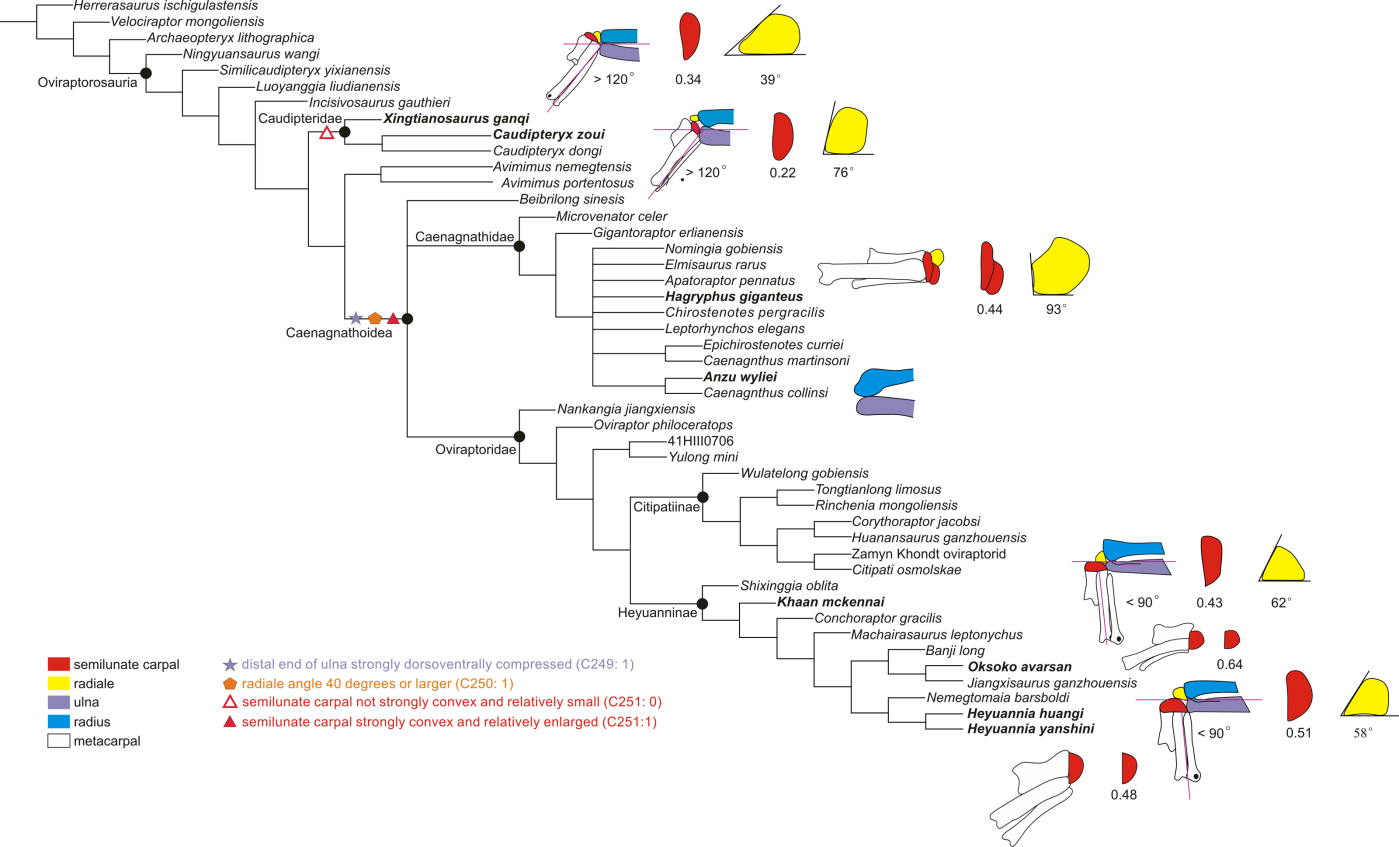
The reduced strict consensus tree showing the major changes of the range of motion of the left wrist during oviraptorosaur evolution. The significant changes of the wrist are shown, including the maximum abduction of the wrist, the shape of the distal ulna and radius, the radiale angle, and the shape of the semilunate carpal (not synapomorphy). The number below the semilunate carpal is the product of multiplying the ratio of the anteroposterior length to the lateromedial length of the semilunate carpal by the ratio of the combined widths of the proximal articular surfaces of the first and second metacarpals to the lateromedial length of the semilunate carpal. The number below the radiale is the radiale angle. The wrist of *Xingtianosaurus* is redrawn from Qiu et al. (2019). The wrist of *Caudipteryx* is redrawn from Zhou & Wang (2000). The wrist of *Hagryphus* is redrawn from Zanno & Sampson (2005). The distal forearm of *Anzu* is redrawn from Lamanna, Sues & Schachner (2014). The wrist of *Khaan* is redrawn from Botelho et al. (2014). The wrist of *Oksoko* is redrawn from Funston et al. (2020). The wrist of *Heyuannia yanshini* is redrawn from Easter (2013). The line drawings are not to scale. The purple lines show the smallest angle of abduction between the manus and the ulna.

Our phylogenetic analysis produced a reduced strict consensus tree based on 45 most parsimonious trees (tree length = 686, retention index = 0.41, consistency index = 0.65). *Anomalipes* and *Ganzhousaurus* were detected as the most unstable taxa by prunnelsen and they were pruned from the reduced strict consensus. The reduced consensus supports *Heyuannia huangi* being closest to *Heyuannia yanshini* (Fig. 4), giving a result similar to the previous results on the evolution of oviraptorosaurs (Funston & Currie, 2016; Lü et al., 2016; Lü et al., 2017; Qiu et al., 2019; Funston et al., 2020). Heyuanninae is the sister taxa of Citipatiinae and is supported by two synapomorphies: 7–8 vertebrae included in the synsacrum in adults (character 113: 2) and anteroposterior length of the pubic peduncle about the same as that of the ischial peduncle (character 148: 0).

**Figure 5:**
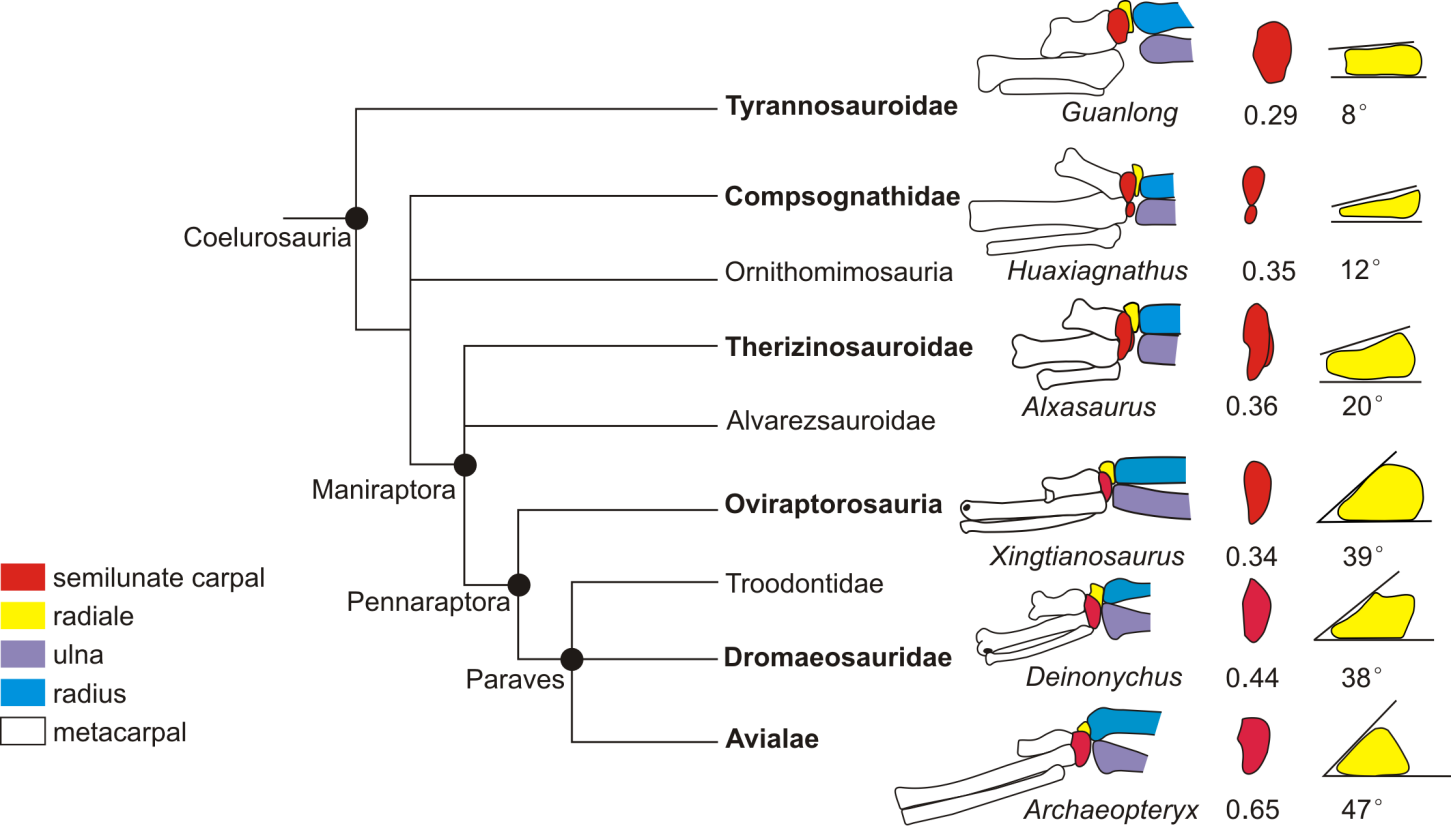
Coelurosaurian phylogeny showing the major changes of the wrist. The coelurosaurian evolutionary tree is based on simplified coelurosaurian phylogeny after Qiu et al. (2019). The number below the semilunate carpal is the product of multiplying the ratio of the anteroposterior length to the lateromedial length of the semilunate carpal by the ratio of the combined widths of the proximal articular surfaces of the first and second metacarpals to the lateromedial length of the semilunate carpal. The number below the radiale is the radiale angle. The wrists of *Guanlong* and *Alxasaurus* are redrawn from Sullivan et al. (2010). The wrist of *Huaxiagnathus* is redrawn from Botelho et al. (2014). The wrist of *Xingtianosaurus* is redrawn from Qiu et al. (2019).The wrist of *Deinonychus* is redrawn from Ostrom (1969). The wrist of *Archaeopteryx* is redrawn from Wellnhofer (2009). The line drawings are not to scale.

The reduced strict consensus tree shows that a strongly dorsoventrally compressed distal ulna (character 249: 1) and a radiale angle of 40 degrees or greater (character 250: 1) are the synapomorphies of Caenagnathoidea. Although the radiale angle is greater than 70 degrees in *Caudipteryx*, the presence of a radiale angle less than 40 degrees in the basal caudipterid *Xingtianosaurus* (Qiu et al., 2019), along with the absence of a strongly convex semilunate carpal and a strongly dorsoventrally compressed distal ulna, indicates that the similarity in the relatively large radiale angle between *Caudipteryx* and Caenagnathoidea is convergently evolved in these taxa. A strongly convex semilunate carpal (character 251: 1) is only found in caenagnathoids among oviraptorosaurs based on the reduced strict consensus tree. A moderately convex semilunate carpal (character 251: 0) is present in the Caudipteridae. It should be noted that the shape of the semilunate carpal of Caudipteridae resembles that of the theropods more basal than the oviraptorosaurs, such as the basal tyrannosaurids, compsognathids, and therizinosauroids (Fig. 5), whereas the shape of the semilunate carpal of Caenagnathoidea resembles that of the paravians (Fig. 5). Whether a strongly convex semilunate carpal or a moderately convex semilunate carpal is the plesiomorphic condition of Oviraptorosuria remains unresolved in this phylogenetic analysis because the semilunate carpal is unknown in most basal oviraptorosaurs except caudipterids (Zhou & Wang, 2000; Qiu et al., 2019).

## Discussion

**Figure 6:**
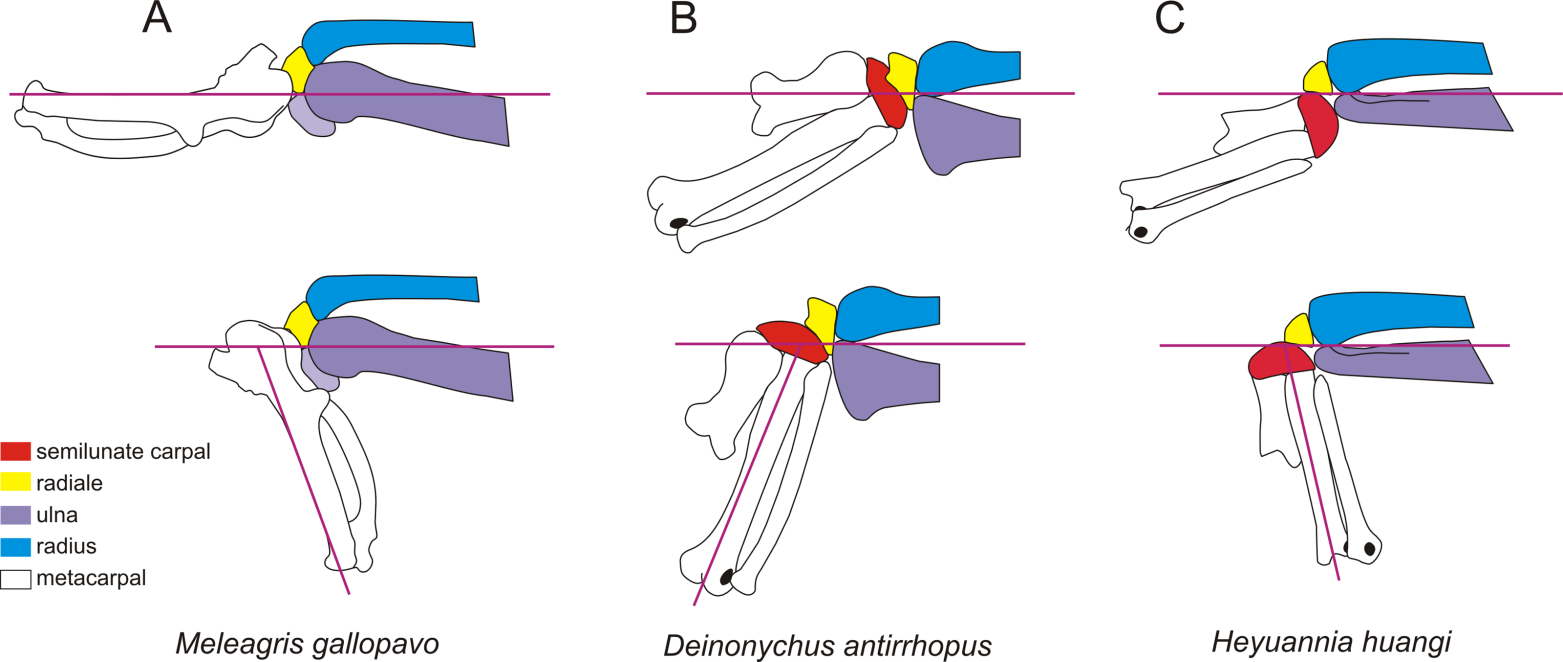
The wrist of turkey, *Meleagris gallopavo* (A), *Deinonychus antirrhopus* (B) and*Heyuannia huangi* (C). The upper wrists are in the maximum adduction, and the lower wrists are in the maximum abduction. The wrist of turkey is modifed from Sullivan et al. (2010). The wrist of *Deinonychus antirrhopus* is modifed from Ostrom (1969). The line drawings are not to scale.

In order to protect the pennaceous feathers on the forelimb from damage, the wrist joint of the extant volant birds is highly specialized, allowing for a large range of abduction in the avian carpus (Sullivan et al., 2010). In extant birds, the smallest angle between the manus and the ulna is less than 60 degrees, even without the movement of radius (Fig. 6A; Carpenter, 2002; Sullivan et al., 2010). The evolution of this function from non-avian theropods to birds has been discussed in the studies examining the range of motion of the wrist in various theropods, including *Deinonychus*, *Bambiraptor*, *Mononykus*, *Acrocanthosaurus* and *Australovenator* (Gishlick, 2001; Carpenter, 2002; Senter & Robins, 2005; Senter, 2005; Senter, 2006; White et al., 2015). Compared with other theropods, the wrist of oviraptorids is obviously specialized. The distal end of radius of oviraptorids is strongly ventrally or dorsoventrally expanded, while the distal end of ulna is strongly dorsoventrally compressed. In the lateral view, the distal end of ulna is much narrower than the distal end of the radius (Longrich, Currie & Dong, 2010; Balanoff & Norell, 2012; Fanti, Currie & Badamgarav, 2012; Norell et al., 2018; Funston et al., 2020; Wei et al., 2022). In birds and other non-avian theropods, the distal end of the ulna is higher than or subequal to the distal end of the radius (Gishlick, 2001; Carpenter, 2002; Senter, 2006). However, there is no study that examines the range of motion of the specialized wrist of oviraptorids. The change in the range of motion of the wrist resulting from the change of the shape of the wrist in oviraptorids is discussed here based on the comparison between the wrist of other non-avian pennaraptorans and the preserved elements of the wrist of *Heyuannia huangi*.

The shape and arrangement of the carpal in *Heyuannia huangi* more closely resemble those of non-avian paravians rather than extant birds (Fig. 6). The semilunate carpal is relatively enlarged and not fused with metacarpals. The strongly convex proximal surface of the semilunate carpal possesses a deep transverse groove and a developed trochlea (Hutson & Hutson, 2014; Xu, Han & Zhao, 2014). The proximal surface of the radiale possesses a concavity at its center in order to contact the radius. It can be easily distinguished from the radiale of extant birds whose proximal surface possesses a sharp ridge, dividing the surface into two facets for contacting the radius and ulna, respectively (Livezey & Zusi, 2006; Mayr, 2014). Since the radiale contacts only the radius in *Heyuannia huangi*, other oviraptorids and non-avian paravians, the center of rotation of the wrist abduction is located higher than or near the joint of the ulna and radius on the lateromedial axis. In extant birds, the radiale contacts both the radius and ulna, causing the center of rotation of the wrist abduction to be located at the center of the distal ulna on the lateromedial axis.

Although the oviraptorids and non-avian paravians share a similar structure on the articular faces of the carpus, the specialized distal end of the forearm in *Heyuannia huangi* indicates a different movement mode of its wrist. In dromaeosaurids, the rotation of the hand is restricted by the dorsoventrally expanded distal ulna because the center of rotation of the wrist abduction is located higher than or near the joint of the ulna and radius on the lateromedial axis. So, the smallest angle between the manus and the ulna should not be less than 100 degrees in dromaeosaurids (Fig. 6B; Gishlick, 2001; Carpenter, 2002; Senter, 2006). Although the distal ulna is also higher than the distal radius in extant birds, it does not hinder the movement of their manus. This is because the center of rotation of the wrist abduction is situated at the center of the distal ulna on the lateromedial axis. The distal end of radius of *Heyuannia huangi* is strongly dorsoventrally expanded, while the distal end of ulna is strongly dorsoventrally compressed. In the lateral view, the distal end of ulna is much narrower than the distal end of radius, and is even narrower than the proximal surface of the semilunate carpal. The specialized condition observed in *Heyuannia huangi*, where the distal end of the ulna is narrower than the proximal surface of the semilunate carpal, differs from the typical morphology seen in most theropods. In many theropods, the distal end of the ulna is either similar in height to the distal radius or higher than the distal radius, and the semilunate carpal is typically narrower than the distal ulna (Gishlick, 2001; Carpenter, 2002). The analysis of joint movement indicates that although the center of rotation of the wrist is still located at the joint of the ulna and radius on the lateromedial axis as in other non-avian pennaraptorans, the flat distal ulna in *Heyuannia huangi* would not impede the rotation of the manus. The smallest angle between the manus and the ulna might be less than 90 degrees in *Heyuannia huangi* (Fig. 6C). This suggests that the range of motion between the manus and forearm in *Heyuannia huangi* is different from that in other non-avian theropods, where this angle is typically larger. Based on the phylogenetic result, a strongly dorsoventrally compressed distal ulna and a larger radiale angle are the synapomorphies of Caenagnathoidea, and a strongly convex semilunate carpal is found only in caenagnathoids among oviraptorosaurs. The shapes of the carpal and forearm in the oviraptorid specimens with well-preserved forelimb are similar to that in in *Heyuannia huangi* (Balanoff & Norell, 2012; Wei et al., 2022), which supports a development wrist abduction. A strongly compressed distal end of ulna is also present in caenagnathids (Makovicky & Sues, 1998; Lamanna, Sues & Schachner, 2014). Although the carpals are poorly preserved in most known caenagnathids, the well preserved carpal of *Hagryphus* possesses many features similar to those of the oviraptorids, including the radiale angle greater than 40 degrees and a strongly convex semilunate carpal. These similarities suggest that a development wrist abduction should be a body plan of Caenagnathoidea rather than Oviraptoridae.

Among oviraptorids, the morphology of the wrist of caudipterids, which are regarded as the basal oviraptorosaurs in the phylogenetic result (Fig. 4), does not indicate a significant capacity for wrist abduction similar to that in the derived oviraptorosaurs. Although some caudipterids possess a large radiale angle (Sullivan et al., 2010), the semilunate carpal of caudipterids is not strongly convex, and the distal end of ulna and radius of caudipterids share a similar height as in other theropods. In addition, the radiale and semilunate carpal of caudipterids are relatively small, with their combined width being smaller than the craniocaudally height of the distal end of either the ulna or radius. Therefore, the limited space between the manus and the forearm of caudipterids suggests that there is no enough space for a large wrist abduction. The smallest angle between the manus and the ulna is estimated to be greater than 120 degrees. The morphology of the wrist combined with the phylogenetic result of oviraptorosaurs indicates a functional convergence in the wrist of the oviraptorids and extant birds.

## Conclusions

The wrist of *Heyuannia huangi* is described in detail and its phylogenetic position has been confirmed by the modified oviraptorosaurian phylogenetic matrix. *Heyuannia huangi* and other oviraptorids possess a specialized wrist, with a strongly convex semilunate carpal, a radiale angle larger than 40 degrees, and a distal ulna that is strongly dorsoventrally expanded to be plate-like. The phylogenetic result indicates that a strongly dorsoventrally compressed distal ulna and a larger radiale angle are the synapomorphies of Caenagnathoidea, and a strongly convex semilunate could be found only in caenagnathoids among oviraptorosaurs. The morphology of its wrist indicates that although the center of rotation of the wrist is located at the joint of the ulna and radius on the lateromedial axis, the flat distal ulna cannot hinder the rotation of the manus, which is different from the condition in other non-avian pennaraptorans. The smallest angle between the manus and the ulna could be less than 90 degrees, similar to extant birds rather than most non-avian theropods. The morphology of the wrist, combined with the phylogenetic result of oviraptorosaurs, indicates a functional convergence in the wrist of the oviraptorids and extant birds.

## Supplemental Information

10.7717/peerj.17669/supp-1 Supplemental Information 1 Character list of Oviraptorosauria and Datamatrix of Oviraptorosauria in TNT format

## Additional Information and Declarations

### Data Availability

The following information was supplied regarding data availability: The raw measurements are available in the Supplementary File.
