# Supplementary material for: The osteology of the wrist of Heyuannia huangi (Oviraptorosauria) and its implications for the wrist folding mechanism
Source: PeerJ. 2024 Jul 10;12:e17669. doi: 10.7717/peerj.17669 (PMC13037537; doi:10.7717/peerj.17669)
Supplement: Supplemental Information 1 [file peerj-12-17669-s001.docx]

APPENDIX

Character list of Oviraptorosauria

1. Ratio of the preorbital skull length to the basal skull length: 0.6 or more (0); less than 0.6 (1)
2. Pneumatized crest-like prominence on the skull roof: absent (0) present (1)
3. Ratio of the width (across premaxilla-maxilla suture) of the snout to its length: less than 0.3 (0); 0.3-0.4 (1); more than 0.4 (2)
4. Ratio of the length of the tomial margin of the premaxilla to the premaxilla height (ventral to the external naris): 1.0-1.4 (0); more than 1.7 (1); 0.7 or less (2)
5. Inclination of the anteroventral margin of the premaxilla relative to the horizontally positioned ventral margin of the jugal: vertical (0); posterodorsal (1); anterodorsal (2)
6. Ventral projection of the premaxilla below the ventral margin of the maxilla: absent (0); present (1)
7. Ventral projection of the premaxilla below ventral margin of the maxilla: small (0); significant (1)
8. Share of the premaxilla (ventral) in the basal skull length: 0.10 or less (0); 0.12 or more (1)
9. Pneumatization of the premaxilla: absent (0); present (1)
10. Ratio of the length of the maxilla (in lateral view) to the basal skull length: 0.4–0.7 510 (0); less than 0.4 (1)
11. Subantorbital portion of the maxilla: not inset medially (0); inset medially (1)
12. Palatal shelf of the maxilla with two longitudinal ridges and a tooth-like ventral process: absent (0); present (1)
13. Ventral margins of maxilla and jugal: margins form a straight line (0); the ventral margin of the maxilla slopes anteroventrally, its longitudinal axis at an angle of ca. 120° to the longitudinal axis of the jugal (1)
14. Rim around antorbital fossa: well pronounced (0); poorly delimited (1)
15. Antorbital fossa: bordered anteriorly by the maxilla (0); bordered anteriorly by the premaxilla (1)
16. Accessory maxillary fenestrae: absent (0); at least one accessory fenestra present (1)
17. Nasal along midline: longer than frontal (0); shorter than or as long as the frontal
18. Nasals: separate (0); fused (1)
19. Subnarial process of the nasal: long (0); short (1)
20. Shape of the narial opening: longitudinally oval (0); teardrop-shaped, slightly longer than wide much longer than wide (1)
21. Nasal recesses: absent (0); present (1)
22. External naris position relative to the antorbital fossa naris and fossa: widely separated (0); posterior margin of the naris reaching the fossa (1) overlapping anterodorsally most of the fossa (2)
23. Ventral margin of the external naris: at the level of the maxilla (0); dorsal to the maxilla (1)
24. Prefrontal: present (0); absent or fused with the lacrimal (1)
25. Lacrimal shaft: not projecting outward beyond the orbital plane and lateral surface of the snout (0); the middle part of the shaft projecting laterally to form a flattened transverse bar in front of the eye (1)
26. Lacrimal recesses: absent (0); present (1)
27. Ratio of the length of the orbit to the length of the antorbital fossa: 0.7-0.9 (0); 1.2 or more (1)
28. Ratio of the length of the parietal to the length of the frontal: 0.6 or less (0); 1.0 or more (1)
29. Pneumatization of the skull roof bones: absent (0); present (1)
30. Sagittal crest along the interparietal contact: absent (0); present (1)
31. Supratemporal fossa: invading the frontal (0); not invading the frontal (1)
32. Infratemporal fenestra: dorsoventrally elongate, narrow anteroposteriorly (0); subquadrate, its anteroposterior length comparable to the orbital length (1)
33. Pneumatization of the squamosal: absent (0); present (1)
34. Cotyle-like incision on the ventrolateral margin of the squamosal (for reception of the dorsal end of the ascending process of the quadratojugal): absent (0); present (1)
35. Ventral ramus of the jugal deep dorsoventrally and flattened lateromedially (0); shallow dorsoventrally or rod-shaped (1)
36. Jugal process of the postorbital: not extending ventrally below two-thirds of the orbit height (0); long, extending ventrally close to the base of the postorbital process of the jugal (1)
37. Postorbital process of the jugal: posterodorsally inclined (0); perpendicular to the ventral ramus of the jugal (1)
38. Postorbital process of the jugal: present (0); absent (1)
39. Jugal-postorbital contact: present (0); absent (1)
40. Quadratojugal process of the jugal in lateral view: forked (0); not forked (1); fused with the quadratojugal (2)
41. Quadratojugal-squamosal contact: absent (0); present (1)
42. Ascending (squamosal) process of the quadratojugal: bordering ca. the ventral half, or less, of the infratemporal fenestra (0); bordering the ventral two-thirds or more of the infratemporal fenestra (1);
43. Ascending (squamosal) process of the quadratojugal: present (0); absent (1)
44. Angle between the ascending and jugal processes of the quadratojugal: ca. 90° (0); less than 90° (1)
45. Quadrate process of the quadratojugal: well developed, extending posteriorly or posteroventrally beyond the posterior margin of the ascending process (0); not extending beyond the posterior margin of the ascending process (1)
46. Dorsal part of the quadrate: erect (0); bent backward (1)
47. Otic process of the quadrate: articulating only with the squamosal (0); articulating with the squamosal and the lateral wall of the braincase (1)
48. Pneumatization of the quadrate: absent (0); present (1)
49. Lateral accessory process on the distal end of the quadrate for articulation with the quadratojugal: absent (0); present (1)
50. Lateral cotyle for the quadratojugal on the quadrate: absent (0); present (1)
51. Mandibular condyles of quadrate: posterior to the occipital condyle (0); in the same vertical plane as the occipital condyle (1); anterior to the occipital condyle (2)
52. Nuchal transverse crest: pronounced (0); not pronounced (1)
53. Occiput position in relation to the ventral margin of the jugal-quadratojugal bar: about perpendicular (0); inclined anterodorsally (1)
54. Paroccipital process: directed laterally (0); directed ventrally (1)
55. Foramen magnum: smaller than or equal in size to the occipital condyle (0); larger than the occipital condyle (1)
56. Basal tubera: modestly pronounced (0); well developed, widely separated (1)
57. Pneumatization of the basisphenoid: weak or absent (0); extensive (1)
58. Basipterygoid processes: well developed (0); strongly reduced (1
59. Basipterygoid processes: present (0); absent (1)
60. Parasphenoid rostrum: horizontal or anterodorsally directed (0); sloping anteroventrally (1)
61. Depression in the periotic region: absent (0); present (1)
62. Pneumatization of the periotic region: absent or weak (0); extensive (1)
63. Quadrate ramus of the pterygoid: distant from the braincase wall (0); overlapping the braincase (1)
64. Pterygoid basal process for contact with the basisphenoid: absent (0); present (1)
65. Ectopterygoid position: lateral to the pterygoid (0); anterior to the pterygoid (1)
66. Ectopterygoid contacts with the maxilla and lacrimal: absent (0); present (1)
67. Ectopterygoid: short anteroposteriorly with a hook-like jugal process (0); elongate, shaped like a Viking ship, without a hook-like process (1)
68. Massive pterygoid-ectopterygoid longitudinal bar: absent (0); present (1)
69. Palate extending below the cheek margin: absent (0); present (1)
70. Palatine: tetraradiate or trapezoidal (0); triradiate, without a jugal process (1); developed in horizontal, longitudinal, and transverse planes perpendicular to each other (2)
71. Pterygoid wing of the palatine: dorsal to the pterygoid (0); ventral to the pterygoid
72. Maxillary process of the palatine: shorter than the vomeral process (0); longer than the vomeral process (1)
73. Vomer: distant from the parasphenoid rostrum (0); approaching or in contact with the parasphenoid rostrum (1)
74. Suborbital (ectopterygoid-palatine) fenestra: well developed (0); closed or reduced
75. Jaw joint: distant from the midline of the skull (0); close to the skull midline (1)
76. Movable intramandibular joint: present (0); suppressed (1)
77. Mandibular symphysis: loose (0); tightly sutured (1); fused (2)
78. Extended symphyseal shelf at the mandibular symphysis: absent (0); present (1)
79. Downturned symphyseal portion of the dentary: absent (0); present (1)
80. U-shaped mandibular symphysis: absent (0); present (1)
81. Ratio of the length of the retroarticular process to the total mandibular length: less than 0.05 or the process absent (0); ca. 0.10 (1)
82. Dentary: elongate (0); proportionally short and deep, with maximum depth of dentary between 25% and 50% of dentary length (with length measured from the tip of the jaw to the end of the posterodorsal process) (1); extremely short and deep, with maximum depth 50% or more of dentary length (2)
83. Ratio of the height of the external mandibular fenestra to the length of the fenestra: 0.2-0.5 (0); 0.7-1.0 (1)
84. External mandibular fenestra: present (0); absent (1)
85. Ratio of the length of the external mandibular fenestra to total mandibular length: 0.15-0.20 (0); not more than 0.10 or fenestra absent (1); 0.25 or more (2)
86. Process of the surangular dividing the external mandibular fenestra: absent (0); short and broad (1); elongate and spike-like (2)
87. Co-ossification of the articular with the surangular: absent (0); present (1)
88. Mandibular rami in dorsal view: straight (0); laterally bowed at midlength (1)
89. Anterodorsal margin of dentary in lateral view: straight (0); concave (1); broadly concave (2)
90. Posterior margin of the dentary: incised, producing two posterior processes (0); oblique (1)
91. Posterodorsal process of the dentary: long and shallow present (0); absent (1)
92. Posteroventral process of the dentary shallow and long, extending posteriorly at least to the posterior border of the external mandibular fenestra: absent (0); present (1)
93. Coronoid process: posteriorly positioned and vertically projected (0); anteriorly positioned, near the midpoint of the jaw, with a medially hooked apex (1)
94. Surangular foramen: present (0); absent (1)
95. Mandibular articular facet for the quadrate: comprising the surangular and the articular (0); formed exclusively of the articular (1)
96. Mandibular articular facet for the quadrate: with one or two cotyles (0); convex in lateral view, transversely wide (1)
97. Position of the quadrate articular surface relative to the level of the adjoining dorsal margin of the mandibular ramus: ventral (0); moderately elevated, quadrate articulation grades smoothly into remainder of mandible (1); highly elevated, anterior and posterior margins of quadrate articulation at nearly right angles to remainder of mandible (2)
98. Anterior part of the prearticular: deep, approaching the dorsal margin of the mandible (0); shallow, strap-like, not approaching the dorsal mandibular margin (1)
99. Splenial: subtriangular, approaching the dorsal mandibular margin (0); strap-like, shallow, not approaching the margin (1)
100. Mandibular adductor fossa: anteriorly delimited, occupying the posterior part of the mandible (0); large, anteriorly and dorsally extended, not delimited anteriorly (1)
101. Coronoid bone: well developed (0); strongly reduced (1)
102. Coronoid bone: present (0); absent (1)
103. Premaxillary teeth: present (0); absent (1)
104. Maxillary tooth row: extends at least to the level of the preorbital bar (0); does not reach the level of the preorbital bar (1); maxillary teeth absent (2)
105. Dentary teeth: present (0); absent from tip of jaw but present posteriorly (1); absent (2)
106. Number of cervicals (excluding cervicodorsal): not more than 10 (0); more than 10 (1)
107. Anterior articular facets of the centra in the anterior postaxial cervicals: not inclined or only slightly inclined (0); strongly inclined posteroventrally, almost continuous with the ventral surfaces of the centra (1)
108. Centra of the anterior cervicals: not extending posteriorly beyond their respective neural arches (0); extending posteriorly beyond their respective neural arches (1)
109. Epipophyses on the postaxial cervicals: in the form of a low crest or rugosity (0); prong-shaped (1)
110. Shafts of cervical ribs: longer than their respective centra (0); not longer than their respective centra (1)
111. Lateral pneumatic fossae ("pleurocoels") on the dorsal centra: absent (0); present (1)
112. Ossified uncinate processes on the dorsal ribs: absent (0); present (1)
113. Number of vertebrae included in the synsacrum in adults: not more than 5 (0); 6 (1); 7-8 (2)
114. Sacral spines in adults: unfused (0); fused (1)
115. Lateral pneumatic fossae on the sacral centra: absent (0); present (1)
116. Transition point on the caudals: absent (0); present (1)
117. Number of caudals with transverse processes: 15 or more (0); fewer than 15 (1)
118. Lateral pneumatic fossae on the caudal centra: absent (0); present at least in the anterior part of the tail (1)
119. Neural spines: confined to at least 23 anterior caudals (0); at most 16 anterior caudals (1)
120. Number of caudals: more than 35 (0); 30 or fewer (1)
121. Posterior caudal prezygapophyses: overlapping less than half of the centrum of the preceding vertebra (0); overlapping at least half of the centrum of the preceding vertebra (1)
122. Hypapophyses in the cervicodorsal vertebral region: absent (0); small (1); prominent (2)
123. Posterior hemal arches: deeper than long (0); longer than deep (1)
124. Ratio of the length of the scapula to the length of the humerus: 0.8-1.1 (0); 1.2 or more (1); 0.7 or less (2)
125. Acromion: projecting dorsally (0); projecting anteriorly (1); everted laterally (2)
126. Posteroventral process of the coracoid: absent or short, not extending beyond the glenoid diameter (0); long, posteroventrally extending beyond the glenoid (1)
127. Orientation of the glenoid on the pectoral girdle: posteroventral (0); lateral (1)
128. Deltopectoral crest: low, its width equal to, or smaller than, the shaft diameter (0); expanded, wider than the shaft diameter (1)
129. Extent of the deltopectoral crest (measured from the humeral head to the apex): about the proximal third of the humerus length or less (0); ca. 40%-50% of the humerus length (1)
130. Shaft of the ulna: straight (0); bowed, convex posteriorly (1)
131. Ratio of the length of the radius to the length of the humerus: 0.80 or less (0); 0.85 or more (1)
132. Combined length of manual phalanges III-1 and III-2: greater than the length of phalanx III-3 (0); less than or equal to the length of phalanx III-3 (1)
133. Ratio of the length of metacarpal I to the length of metacarpal II: 0.5 or more (0); less than 0.5 (1)
134. Proximal margin of metacarpal I in dorsal view: straight, horizontal (0); angled due to a medial extent of carpal trochlea (1)
135. Metacarpal II relative to metacarpal III: shorter (0); longer (1); subequal (2)
136. Ratio of the length of metacarpal II to the length of the humerus: 0.4 or less (0); more than 0.4 (1)
137. Ratio of the length of the manus to the length of the humerus plus the radius: 0.50-0.65 (0); more than 0.65 (1); less than 0.50 (2)
138. Ratio of the length of the manus to the length of the femur: 0.3-0.6 (0); more than 0.7 (1)
139. Ratio of the length of the humerus to the length of the femur: 0.50-0.69 (0); 0.70 or more (1)
140. Dorsal margins of opposite iliac blades: well separated from each other (0); close to or contacting each other along their medial sections (1)
141. Dorsal margin of the ilium along the central portion of the blade: straight (0); arched (1)
142. Preacetabular process of the ilium relative to the postacetabular process (lengths measured from the center of the acetabulum): shorter or equal (0); longer (1)
143. Preacetabular process: not expanded or weakly expanded ventrally below the level of the dorsal acetabular margin (0); expanded ventrally well below the level of the dorsal acetabular margin (1)
144. Morphology of the ventral margin of the preacetabular process: cuppedicus fossa absent, margin transversely narrow (0); cuppedicus fossa or a wide shelf present (1); margin flat, wide at least close to the pubic peduncle (2)
145. Anteroventral extension of the preacetabular process: absent (0); present (1)
146. Anteroventral extension of the preacetabular process: with rounded tip (0); hook-like (1)
147. Posterior end of the postacetabular process: truncated or broadly rounded (0); narrowed or acuminate (1)
148. Anteroposterior length of the pubic peduncle: about the same as that of the ischial peduncle (0); distinctly greater than that of the ischial peduncle (1)
149. Dorsoventral extension of the pubic peduncle: level with the ischial peduncle (0); deeper than the ischial peduncle (1)
150. Ratio of the length of the ilium to the length of the femur: 0.50-0.79 (0); 0.80 or more (1)
151. Pelvis: propubic (0); mesopubic (1); opisthopubic (2)
152. Pubic shaft: straight (0); concave anteriorly (1)
153. Pubic foot: anterior and posterior processes about equally long (0); anterior process absent or shorter than the posterior process (1); anterior process longer than the posterior process (2)
154. Posterior margin of the ischial shaft: straight or almost straight (0); distinctly concave (1)
155. Greater trochanter of the femur: weakly separated, or not separated, from the femoral head (0); distinctly separated from the femoral head (1)
156. Anterior and greater trochanters: separated (0); contacting (1)
157. Dorsal extremity of the anterior trochanter: well below the greater trochanter (0); about level with the greater trochanter (1)
158. Fourth trochanter: well developed (0); weakly developed or absent (1)
159. Adductor fossa and the associated anteromedial crest on the distal femur: weak or absent (0); well developed (1)
160. Distal projection of the fibular condyle of the femur beyond the tibial condyle: absent (0); present (1)
161. Ascending process of the astragalus: as tall as wide across the base (0); taller than wide (1)
162. Distal tarsals: not fused with the metatarsus (0); fused with the metatarsus (1)
163. Proximal coossification of metatarsals II-IV: absent (0); present (1)
164. Arctometatarsus: absent (0); present, but only proximal-most extreme of metatarsal III obscured from anterior view in articulated metatarsus (1); present, proximal ~half of metatarsal III obscured from anterior view in articulated metatarsus (2)
165. Length of metatarsal I: constituting more than 50% of metatarsal II length (0); less than 50% of metatarsal II length (1); metatarsal I absent (2)
166. Ratio of the maximum length of the metatarsus to the length of the femur: 0.4-0.6 (0); ca. 0.3 (1); 0.7-0.8 (2)
167. Crenulated tomial margin of the premaxilla: absent (0); present (1)
168. Frontals: flat or weakly arched, not strongly projecting above orbit in lateral view (0); strongly arched, projecting well above orbit in lateral view to contribute to nasal-frontal crest (1)
169. Exoccipital: short, weakly projecting (0); strongly projects ventrally beyond squamosal in lateral view, approaching ventral end of the quadrate (1)
170. Dentary posterodorsal ramus: straight or weakly curved (0); strongly bowed dorsally (1)
171. Prominent symphyseal process on posteroventral surface of dentary symphysis: absent (0); present (1)
172. Dentary: anteroventral margin in lateral view straight or weakly downturned (0); strongly downturned (1)
173. Lateral surface of dentary: smooth (0); bearing a deep fossa, sometimes with associated pneumatopore (1)
174. Angular: contributes extensively to the border of the external mandibular fenestra (0); largely excluded from the border of the external mandibular fenestra by the surangular (1)
175. Surangular with an anteroposteriorly elongate flange on the ventral edge: absent (0); present (1)
176. External mandibular fenestra: elongate (0); height subequal to length (1)
177. Dentary contribution to external mandibular fenestra: no more than 50% length of dentary (0); exceeds 50% length of dentary (1)
178. Metacarpal I expanded ventrally to cover ventral surface of metacarpal II: absent (0); present (1)
179. Unguals of manual digits II and III: strongly curved (0); weakly curved (1)
180. Manual phalanx I-1: slender (0); more robust than II-1 (1); more than 200% diameter of II-1 (2)
181. Manual phalanx III-3: longer than phalanx III-2 (0); does not exceed length of III-2 (1)
182. Manual phalanx II-2: longer than II-1 (0); subequal to or slightly shorter than II-1 (1); distinctly shorter than II-1 (2)
183. Manual digit II: elongate, with combined lengths of manual phalanges II-1 and II-2 longer than metacarpal II (0); combined lengths of manual phalanges II-1 and II-2 subequal to metacarpal II (1)
184. Ischium strongly bent posteriorly at midshaft distal end forms an angle of at least 60 with proximal end absent (0); present (1)
185. Metatarsus: elongate (0); short, length does not exceed 300% of proximal width
186. Ilium: tall (0); low and anteroposteriorly elongate, height less than 25% of length (1)
187. Anterior blade of ilium shallower than posterior blade: absent (0); present (1)
188. External naris: placed anteriorly (0); extends posteriorly, with posterior end lying above antorbital fenestra (1)
189. Premaxillae, nasal processes anteroposteriorly expanded and mediolaterally compressed to form a bladelike internarial bar: absent (0); present (1)
190. Dentary, anterodorsal tip of beak: projects upwards (0); anterodorsally, tip of beak projecting at an angle of 45° or less relative to the ventral margin of the symphysis (1)
191. Dentary symphysis with interior surface bearing vascular grooves and associated foramina: absent (0); present (1)
192. Dentary symphysis bearing an hourglass-shaped ventral depression: absent (0); present (1)
193. Meckelian groove terminates on the inside of the dentary (0); on the ventral surface of the symphysis
194. Lingual triturating shelf of dentary: absent (0); present (1)
195. Dentary, symphyseal ridges inside the tip of the beak: absent (0); present but weakly developed (1); present and well developed (2)
196. Dentary, lingual ridges inside the lateral occlusal surface of beak: absent (0); present (1)
197. Posteroventral process of dentary: straight (0); bowed ventrally (1)
198. Dentaries pneumatized: absent (0); present (1)
199. Dentary: participates in dorsal border of the external mandibular fenestra (0); excluded from dorsal border of external mandibular fenestra by anterior extension of the surangular (1)
200. Dentary: participates in ventral border of external mandibular fenestra (0); excluded from ventral border of external mandibular fenestra by anterior extension of the angular (1)
201. Surangular and angular divided by posterior extension of the external mandibular fenestra: absent (0); present (1)
202. Posterior end of the surangular: deep (0); shallow, subequal to or shallower than angular (1)
203. Surangular: deep anteriorly (0); strap-like (1)
204. Retroarticular process extends: posteriorly (0); posteroventrally (1); posterolaterally (2)
205. Metacarpal I: proportionately broad (0); long and slender, diameter 20% of length (1)
206. Manual phalanx I-1: longer than II-2 (0); subequal to II-2 (1); shorter than II-2 (2)
207. Ischiadic peduncle of pubis with prominent medial fossa: absent (0); present (1)
208. Ischium, obturator process located: distally (0); at midshaft of ischium (1)
209. Anterior margin of obturator process: straight or convex (0); distinctly concave (1)
210. Accessory trochanter of femur: weakly developed (0); prominent, subrectangular flange or finger-like process (1)
211. Metatarsal III: with an ovoid or subtriangular cross section (0); anteroposteriorly flattened, with a concave posterior surface (1)
212. Paroccipital process: elongate and slender, with dorsal and ventral edges nearly parallel (0); short and deep with convex distal end (1)
213. Mandibular articulation surface: as long as ventral end of quadrate (0); twice or more as long as quadrate surface, allowing anteroposterior movement of mandible (1)
214. Sternum, distinct lateral xiphoid process posterior to costal margin: absent (0); present (1)
215. Anterior edge of sternum: grooved for reception of coracoids (0); without grooves (1)
216. Deltopectoral crest: large and distinct, proximal end of humerus quadrangular in anterior view (0); less pronounced, forming an arc rather than being quadrangular (1)
217. Ischium: more than two-thirds of pubis length (0); two-thirds or less of pubis length (1)
218. Lateral ridge of femur: absent or represented (0); only by faint rugosity distinctly raised from shaft, mound-like (1)
219. Surangular, distinct groove on dorsal surface: present (0); absent (1)
220. Vomer, position: level with other palatal elements (0); ventral to other palatal elements (1)
221. Calcaneum: excludes astragalus from reaching lateral margin of tarsus (0); small process of astragalus protrudes through a circular opening in edge of calcaneum to reach lateral margin of tarsus (1)
222. Depression on lateral surface of dentary immediately anterior to external mandibular fenestra: absent (0); present (1)
223. Groove on ventrolateral edge of angular to receive posteroventral branch of dentary: absent (0); present (1)
224. Posteroventral branch of dentary twisted so that lateral surface of branch faces somewhat ventrally: absent (0); present (1)
225. Premaxilla, large, presumably pneumatic foramen at anteroventral corner of narial fossa: absent (0); present (1)
226. Accessory opening at anterodorsal extreme of snout: absent (0); present (1)
227. Development of symphyseal shelf of mandible: limited, anteroposterior length of mandibular symphysis (as measured on midline) less than 20% total anteroposterior length of mandible (0); intermediate, length of symphysis greater than 20% but less than 25% length of mandible (1); extensive, length of symphysis greater than 25% mandibular length (2)
228. Prominent flange or shelf arising from lateral surface of dentary: absent (0); present (1)
229. Base of retroarticular process: considerably wider mediolaterally than tall (0); dorsoventrally approximately as wide as tall (1); considerably taller than wide (2)
230. Posterior-most caudal vertebrae fused, forming a pygostyle-like structure: absent (0); present (1)
231. Humeral shaft: straight or nearly straight (0); strongly bowed laterally (1)
232. Proximodorsal "lip" on manual unguals: weak (i.e., continuous or nearly continuous with remainder of dorsal surface of ungual) and/or absent (0); prominent ("set off" from remainder of dorsal surface by distinct change in slope immediately distal to "lip") (1)
233. Pubic process of ischium, "hooked" anterodistal extension: absent (0); present (1)
234. Posterodistal margin of obturator process: straight (0); distinctly concave, apex of obturator process angled distally (1)
235. Proximolateral edge of metatarsal IV attenuated into pointed process: absent (0); present (1)
236. Frontal anteriorly divided by slot for nasal and possibly lacrimal: absent (0); present (1)
237. Infradiapophyseal infraprezygapophyseal and infrapostzygapophyseal fossae on cervical and dorsocervical vertebrae: one or more absent (0); all three present (1)
238. Ratio of the length of the metatarsus to the length of the tibia: <0.5 (0); >0.5 (1)
239. Tibia ratio of the transverse width of the distal condyles to the length: 0.20 or greater (0); <0.20 (1)
240. Ratio of minimum transverse width to length of tarsometatarsus: >0.20 (0); <0.20 (1)
241. Fusion of distal tarsals III and IV at maturity: absent (0); present (1)
242. 'Hook-like' posterodorsal process of distal tarsal IV: absent (0); present (1)
243. Posterior protuberance on proximal end of tarsometatarsus caused by coossification of distal tarsals III and IV, plus MT II, III and IV: absent (0); present (1)
244. Anterior margin of metatarsal V in lateral view: straight or slightly curved (0); tightly curved (1)
245. Concavity on posterior surface of tarsometatarsus in cross section: absent or shallow (0); prominent and deep (1)
246. Transverse groove between flexor tubercle and proximal articular surface of manual ungual I-2: absent (0); present (1)
247. Ratio of width of metacarpal III to metacarpal II: more than 0.5 (0); less than 0.5 (1) (Qiu et al., 2019)
248. Ligament pit on manual phalanges: small and dorsally located (0); large and covering most distal end (1) (Qiu et al., 2019)
249. Distal end of ulna: (0) not strongly dorsoventrally compressed; strongly dorsoventrally compressed (1)
250. Radiale: smaller than 40 degrees (0); 40 degree or larger (1)
251. Semilunate carpal, the product of multiplying the ratio of the anteroposterior length to the lateromedial length of the semilunate carpal by the ratio of the combined widths of the proximal articular surfaces of the first and second metacarpals to the lateromedial length of the semilunate carpal: (0) <0.4, semilunate carpal not strongly convex; (1) >0.4, semilunate carpal strongly convex.

Datamatrix of Oviraptorosauria in TNT format

nstates 8 ;

xread 'Data saved from TNT'

251 48

&[num]

Herrerasaurus_ischigualastensis 000100?000000000000000000000000000000000000000000000000000000000000000000000000000001000000000000000000000001?0000000000100?0000101??000100000000?0000101000000000000100000?000?0000110110100??000??0000101??00??0000??00?1000?000000?00000?????000?0000000

Velociraptor_mongoliensis 000200?0000001010010100101000100000000001000000000100001000000000000000000000000000000000110000000000001001010111101101011111?1101011120211000011011112020011100010010000000000?0000000111000?0000??0010000100000000010011?000?00000000000000???0000001000?

Archaeopteryx_lithographica 000?10?000000101101001100010001?1000?1110??0?1??0020????000????0000001?1???1000010?10000011?01000000?10100??001?00?110110010111001111111111011010?111020201?0100?1101200?0000?0?0?00000101000?0000??001?000000?0100100?111??00?00000?0000?00????00000010001

Incisivosaurus_gauthieri 100210?101000011?0111210011?01?00?00000?100010?0??200111?10?10?01??111?0101?2101110020??00011??11?1110011?????????????????????????????????????????????????????????????00000000100??????????00100000000000000???????1??????00???01000???????0?????????0?????

Caudipteryx_zoui 10?210??01??0?1110?0?21?01?10???011000011100100???????????????????0????????0??1111??2???0001??????11??0220????010????0?1??11???1011?1010000?0112110110?021100???1000121??0???0??0?00?001000?000?00000000?01000?0010??0101????0?00000?0000000??????000011010

Avimimus_portentosus ?0?????????0???????????????10110??1??0021?101??00?101111?10???????????????0?1??111???01?0???11000????11?2100010?110??0???2?12100111?1?????00000?1110?10020101001111222100010000?0???????00??00?00????0??0000??0??1011??11110????????0?0?00100?1111000???00?

Avimimus_nemegtensis ?0?2???01???????011202??????01?0???????2110011100?10111101011011??????????0?2101?1?0????00????????????1?2?????0??????0???2??21000??????????0001211100???2?10100111122?1?00100???????????000100100000?00??????????101???1?1??????0??0?00???1?0?1111100??????

Microvenator_celer ????????????????????????????????????????????????????????????????????????????1111?2?0???12001????????????2???0?1?????????0????1?0011??1????0???1111?10?112?1011101????????0101???0011?????00??0000000?0??????0?1??1?????1?0??11????00??00????1?1?????????1??

Oviraptor_philoceratops 1???????????1?11?????21????????1??10000????0????????????????????????1?????????1?121022??00011???1??1???22???0?1????????????2???101101?212????????????????????????0?????0???001001000000??0??1????????????????0?????????0??11?0?0???0??00???0?????????000???

Rinchenia_mongoliensis 11102??0111110111???1211111111?11110100111000111111?11111?11?111111112111111111112102?01100111111111?1122111011?1010010102?22101000???????011011111101????111111?0????1101?001011????????00111??????00000001????????110???1110?0?0?0?????????????????0?????

Citipati_osmolskae 1120211111111011011112111111111111111001110111?1??11111??101111?1111121?111111111210220110011111111110122??????1????????????????0?101?1??????????????????????????0????11011001011?01010?0??1100000000000000100?????01100?01110?001000100?0?0????00?0?0001??

Zamyn_Khondt_oviraptorid 112021111111101111111211111111111111100111011111111111111?111111111112111111111112102201100111111111?1122111011?1010010102102101011011112101001210100111211111111000111101?001011?0000010??110?0000000000?0100???00?1100??11???001?0??00??00?????????000???

Khaan_mckennai 10201101?1111011?112121111111111?110000111000?111?111111???0??1?????1??????111111210210110011111111???1221110?1????00?0102??210??1101011210?0001100001?1211111?1100011100110010010010101111110?000?001000001000000?011100?1?10101000?0000100????00000000111

Conchoraptor_gracilis 10101101111110111112121111111111111000011100?1111111?1111?11111111111211111111111?102?01100111111111?1122111011?201001010112210111100111210100021000011121111111100011101?????????110???1??11????????????????0??????1?00?011?0?0000011?0??000???00000000???

Machairasaurus_leptonychus ????????????????????????????????????????????????????????????????????????????????????????????????????????2??????????????????????????0011??????????????????????????????????????????111110?????????????????????00?????????????????????????1?????????????01011?

Nemegtomaia_barsboldi 11100101111110111111121111111111?11000010101011?111111?1???11?11111112111111111112102?11100111111?11?1122111011?201????????????????????????10002???00?1????11????0????10111101011?12?11??1111000000010000001000??0??1??1??11?1?1?0001?000??00????????0011??

Heyuannia_huangi ??????????????????????????????????1???????????????????????????????????????????1??21021??1001????1???????21???1?12????1?????221?11000001?20???01?10100111?1???????0???????1?1?1011112?2111?1?????????0?00000100?0?0?011?00?1?11?0???0?00001??????00000001111

Heyuannia_yanshini ????????1???????????????11?????1?1100001110?0111111???111???11?1??111?1???11111112102?01100111111111?1122?11011?201001010102210110000110000100121000011121111111100011?011?10101111212111111100000000000000100?0000?1100001?1??0???0?000010?0000000000011?1

Gigantoraptor_erlianensis ??????????????????????????????????????????????????????????????????????????11211112002011200111112?????1?2??????????0010?0??10?00000?11?1??1????????????1??1111111000?1???0001010000??????????0???0001001110100???0?????1?????110??112?10????????010?01?????

Caenagnathasia_martinsoni ????????????????????????????????????????????????????????????????????????????2101?1?????120??????????????2?100?1???1???????????????????????????????????????10?1???????????0001???0????????????0111111?1?????????????????????????????0????????0??????????????

Leptorhynchos_elegans ???????????????????????????????????????????????????????????????????????????12101?1?0??11200???????1?????2?????????1?????????????????????????0??1??011????????????111?????0001???????????000??0111121?111??????????1??????????1????10??????1??11111101??????

Chirostenotes_pergracilis ??????????????????????????????????????????????????????????????????????????1121011100201120011111211110122?1001??111001010?1??10?????0????1??1111111101???1111111100111???00010100?000102000??01111211111111112?1111?1????01?1111??101??1000?111001011110???

Caenagnathus_collinsi ??????????????????????????????????????????????????????????????????????????11210111002011200111111111?1??2????????????1??????????????????????0?1110?10?10??1111111001?????00010100?0??????00??111112101111111??1??1???????01?0010??201??1??????????0??0?????

Anzu_wyliei ?1??10?1100?1100??????????????????0?0000???????1011??100?10??1??0?011?????112101110020112001111111?1?1122111011?1?10010102012100010????1210??1111?010110211111111001??1?000010100000??02000?0111112111011111??1111111101101?001010212011100?1?1??1?010?01??

Hagryphus_giganteus ???????????????????????????????????????????????????????????????????????????????????????????????????????????????????????????????????0010??????????????????????????????????????????000010??????????????????????2?????????????????????????1?????????????110?11

Elmisaurus_rarus ????????????????????????????1?0?????????????????????????????????????????????2101?1?0????200?????????????2???????1?1??????2??2?1????001????????????????10??111????111?0?0?0001????000010?0????0111121?1??????111??11??????0???1????10???10?11111111101110???

Nomingia_gobiensis ????????????????????????????????????????????????????????????????????????????????????????????????????????????0?1?11100101021????????????????1111110010110011111111??????????????????????2?00???????????????????1111??????10??1????????1??00??1?1????????????

Epichirostenotes_curriei ??????????01?010??????1??????????????????????????????110?10?1??0???????????????????????????????????????2??????1?111??1???2?????????????????????????????021??????????????0??????????????2??????????????????????111??0????1???????????????10??0??????????????

Banji_long 11?010?111???0111102121111111011??111001?????0??11?1111?????????1111121?0111??11?21021??000110??1?11??122?1???????????????????????????????????????????????????????????11111001011??????????110??????0?00000???????????????00?0?000?0???????0???????????????

Caudipteryx_dongi ??????????????????????????????????????????????????????????????????????????????????????????????????????????????01?0?????????????0010?1?10000?011?11011000211?????100012????????????00?001000?????????????????01?10????0??1?????????????000000????00000?11010

Ganzhousaurus_nankangensis ???????????????????????????????????????????????????????????????????????????11111?210?2011001?????111????2???????????0???0????????????????????????????????????????0001????1?001?11???????1????????000?1?10?????????0??????????011???0????????????00000??0???

Jiangxisaurus_ganzhouensis 1??????????1?????????????11???????1?0001????????112?????1?1?????1?01121??011111112102111100111111?????122??10?11??1??1?????221?1110?0120????0?????1???????????????????1??110010111?2?????????111??0?0100000100??????11?0?????0011?00??00??????????????00???

Nankangia_jiangxiensis ???????????????????????????????????????????????????????????????????????????11101?2?0????000?????????????2?????1???1??1??0?0111001?????????01110110111111211111111????????1100??????????2?00??11?????0?0???1????111?????01???1010??00??0?10??0?0????????????

Shixinggia_oblita ??????????????????????????????????????????????????????????????????????????????????????????????????????????????1?2?1??1?????????????????????110000?000?????1????1?????????????????????????01????????????????????????????????????????????????????????????????

Similicaudipteryx_yixianensis ????????????????????????????????????????????????????????????????????????????????????????????????????????????011?0?1?0??1?2???????1????????0?001?111110?????????0??0?12???????????????????00???????????????????????0??????????????????10????00???000?0??????

Wulatelong_gobiensis 11?02101?11?1?1???02121?111?1??1?1110001???00??11????????????????1111????????????????101????1??11?????12??????1???100????2??????????1??????1100?1111111121???????000?11???????????0??????011????????????000????1????110?101????????????0??00????????0?0????

Yulong_mini 102110?1110110110111121010111010?0100001110010?111110110???0????????0?????111111121022011001111111?1??122?100?1????001?10??2??0000101?11110?110?100??0??1?1?0??110001110?11001011?11000?100110??????0?0000010???????1?????1110?01000??00???0????000?0?101??

Huanansaurus_ganzhouensis 112021111111101101?11211111111111?110001110111?11100111?????1???11?11?????11111112102201100111111?????122?11011????????????????001101?112?????????????????????????????11011000011?01000????1?1?0????0100000110?????01??0??1??011??101?01???00???????00?????

Tongtianlong_limosus 102021111111101?011212111111101111110001100001111?21110?????011?????1?????1111111210210110011011??????122111001??01?01??0?02?1001?0???????01??????????1121??????1000?111010001011??????11??111??????01000001???10?101010??1?100010001?0??00000??0??000?????

Corythoraptor_jacobsi 1121111111111001???21211111?1??01?11000???????????2?1???????????????1?????111111?2102???0001??????1???122111011?111??1???2?????001101?11100110111110111121111111?1001111?11001011001010100011100000001000?0?00?1000????110???0110110??01010000?1000000?????

Apatoraptor_pennatus ?????????????????????????????????????????????????????????????????????0?1???1211111002011100111111111?1??21100111?????????2?1211001001120211???1110????????111101?????????00010100000?10??????01?11?11111111110???1??1101?01??011??101?01????1????????010???

Oksoko_avarsan 11?211101111100101001211111111001100000111000111012001110101111111111???0001211112102211100111111??1?1122111011111100101020121010000112020010012100001112111110111001011110101011112?1111101100000000100001100010100100000101010000010000100100000000011??1

Anomalipes_zhaoi ??????????????????????????????????????????????????????????????????????????????????????????????????????????????????????????????????????????????????????????11?10??000?0???????????????????????????????????????????00??????1???????????????????01?????0??????

Ningyuansaurus_wangi 10??????????0?????????????????????????????????????????????????????????????????0???????????????????????0?0?????00???????1???0???00?0?1?2?0?0?100?101000?0?1??????1?0??2?0???0??????0???02000?????????????????0??10????0??1????????????00000?????????????????

Xingtiansaurus_ganqi ???????????????????????????????????????????????????????????????????????????????????????????????????????????????????00011??0????0011?0021010??11211????0??1???10??00?12???????????000??020???????????????????0??10?0????01????????????000000??11?0??0?010000

Luoyanggia_liudianensis ?????????????????????????????????????????????????????????????????????????????000?????????0??????????????2???????????????????????????????????0?1???011????1?????????????????0???????????200????0??000???????????00???????1??????????0????10?????????????????

Beibeilong_sinensis 1?0?????1?11000101?????1?01?0?0???11000?1???????11??????????????11????????11111112002?0?000111112?????112???0?1??????????????????0?????????1111?110111?1??11111??000?0?1??10?0?00???????000??00?0?00??00?101?????00?1?????1???10??0?1??????????0????0??????

41HIII0706 ????????????????????????????????????????????????????????????????????????????????????????????????????????????????????010?0?1?2?01?0?0111??????????????????????????????????????????011000?????????????????????00?????????0?????????????001?????????????1101??

;

Ccode

-[/1 0 -[/1 1 -[/1 2 -[/1 3 -[/1 4

-[/1 5 -[/1 6 -[/1 7 -[/1 8 -[/1 9

-[/1 10 -[/1 11 -[/1 12 -[/1 13 -[/1 14

-[/1 15 -[/1 16 -[/1 17 -[/1 18 -[/1 19

-[/1 20 -[/1 21 -[/1 22 -[/1 23 -[/1 24

-[/1 25 -[/1 26 -[/1 27 -[/1 28 -[/1 29

-[/1 30 -[/1 31 -[/1 32 -[/1 33 -[/1 34

-[/1 35 -[/1 36 -[/1 37 -[/1 38 -[/1 39

-[/1 40 -[/1 41 -[/1 42 -[/1 43 -[/1 44

-[/1 45 -[/1 46 -[/1 47 -[/1 48 -[/1 49

-[/1 50 -[/1 51 -[/1 52 -[/1 53 -[/1 54

-[/1 55 -[/1 56 -[/1 57 -[/1 58 -[/1 59

-[/1 60 -[/1 61 -[/1 62 -[/1 63 -[/1 64

-[/1 65 -[/1 66 -[/1 67 -[/1 68 -[/1 69

-[/1 70 -[/1 71 -[/1 72 -[/1 73 -[/1 74

-[/1 75 -[/1 76 -[/1 77 -[/1 78 -[/1 79

-[/1 80 -[/1 81 -[/1 82 -[/1 83 -[/1 84

-[/1 85 -[/1 86 -[/1 87 -[/1 88 -[/1 89

-[/1 90 -[/1 91 -[/1 92 -[/1 93 -[/1 94

-[/1 95 -[/1 96 -[/1 97 -[/1 98 -[/1 99

-[/1 100 -[/1 101 -[/1 102 -[/1 103 -[/1 104

-[/1 105 -[/1 106 -[/1 107 -[/1 108 -[/1 109

-[/1 110 -[/1 111 -[/1 112 -[/1 113 -[/1 114

-[/1 115 -[/1 116 -[/1 117 -[/1 118 -[/1 119

-[/1 120 -[/1 121 -[/1 122 -[/1 123 -[/1 124

-[/1 125 -[/1 126 -[/1 127 -[/1 128 -[/1 129

-[/1 130 -[/1 131 -[/1 132 -[/1 133 -[/1 134

-[/1 135 -[/1 136 -[/1 137 -[/1 138 -[/1 139

-[/1 140 -[/1 141 -[/1 142 -[/1 143 -[/1 144

-[/1 145 -[/1 146 -[/1 147 -[/1 148 -[/1 149

-[/1 150 -[/1 151 -[/1 152 -[/1 153 -[/1 154

-[/1 155 -[/1 156 -[/1 157 -[/1 158 -[/1 159

-[/1 160 -[/1 161 -[/1 162 -[/1 163 -[/1 164

-[/1 165 -[/1 166 -[/1 167 -[/1 168 -[/1 169

-[/1 170 -[/1 171 -[/1 172 -[/1 173 -[/1 174

-[/1 175 -[/1 176 -[/1 177 -[/1 178 -[/1 179

-[/1 180 -[/1 181 -[/1 182 -[/1 183 -[/1 184

-[/1 185 -[/1 186 -[/1 187 -[/1 188 -[/1 189

-[/1 190 -[/1 191 -[/1 192 -[/1 193 -[/1 194

-[/1 195 -[/1 196 -[/1 197 -[/1 198 -[/1 199

-[/1 200 -[/1 201 -[/1 202 -[/1 203 -[/1 204

-[/1 205 -[/1 206 -[/1 207 -[/1 208 -[/1 209

-[/1 210 -[/1 211 -[/1 212 -[/1 213 -[/1 214

-[/1 215 -[/1 216 -[/1 217 -[/1 218 -[/1 219

-[/1 220 -[/1 221 -[/1 222 -[/1 223 -[/1 224

-[/1 225 -[/1 226 -[/1 227 -[/1 228 -[/1 229

-[/1 230 -[/1 231 -[/1 232 -[/1 233 -[/1 234

-[/1 235 -[/1 236 -[/1 237 -[/1 238 -[/1 239

-[/1 240 -[/1 241 -[/1 242 -[/1 243 -[/1 244

-[/1 245 -[/1 246 -[/1 247 -[/1 248 -[/1 249

-[/1 250 ;

;

Ancstates

-0 -1 -2 -3 -4 -5 -6 -7 -8 -9

-10 -11 -12 -13 -14 -15 -16 -17 -18 -19

-20 -21 -22 -23 -24 -25 -26 -27 -28 -29

-30 -31 -32 -33 -34 -35 -36 -37 -38 -39

-40 -41 -42 -43 -44 -45 -46 -47 -48 -49

-50 -51 -52 -53 -54 -55 -56 -57 -58 -59

-60 -61 -62 -63 -64 -65 -66 -67 -68 -69

-70 -71 -72 -73 -74 -75 -76 -77 -78 -79

-80 -81 -82 -83 -84 -85 -86 -87 -88 -89

-90 -91 -92 -93 -94 -95 -96 -97 -98 -99

-100 -101 -102 -103 -104 -105 -106 -107 -108 -109

-110 -111 -112 -113 -114 -115 -116 -117 -118 -119

-120 -121 -122 -123 -124 -125 -126 -127 -128 -129

-130 -131 -132 -133 -134 -135 -136 -137 -138 -139

-140 -141 -142 -143 -144 -145 -146 -147 -148 -149

-150 -151 -152 -153 -154 -155 -156 -157 -158 -159

-160 -161 -162 -163 -164 -165 -166 -167 -168 -169

-170 -171 -172 -173 -174 -175 -176 -177 -178 -179

-180 -181 -182 -183 -184 -185 -186 -187 -188 -189

-190 -191 -192 -193 -194 -195 -196 -197 -198 -199

-200 -201 -202 -203 -204 -205 -206 -207 -208 -209

-210 -211 -212 -213 -214 -215 -216 -217 -218 -219

-220 -221 -222 -223 -224 -225 -226 -227 -228 -229

-230 -231 -232 -233 -234 -235 -236 -237 -238 -239

-240 -241 -242 -243 -244 -245 -246 -247 -248 -249

-250 ;

xgroup

;

agroup

;

taxcode

+0 +1 +2 +3 +4 +5 +6 +7

+8 +9 +10 +11 +12 +13 +14 +15

+16 +17 +18 +19 +20 +21 +22 +23

+24 +25 +26 +27 +28 +29 +30 +31

+32 +33 +34 +35 +36 +37 +38 +39

+40 +41 +42 +43 +44 +45 +46 +47

;

blocks 0;

proc/;
